# Prognostic utility of macrophage polarization (CD68/CD163 ratio) in Egyptian JAK2 positive myeloproliferative neoplasm patients: a single center study

**DOI:** 10.1186/s13000-025-01727-x

**Published:** 2025-11-14

**Authors:** Shirihan Mahmoud Anwar Mahgoub, Marwa Salah Mohamed Yehia, Aya Mohammed Adel Arafat

**Affiliations:** 1 Lecturer of Clinical and Chemical Pathology, Faculty of Medicine, Cairo University, Cairo , Egypt; 2 Lecturer of Internal Medicine, Hematology, Faculty of Medicine, Cairo University, Cairo , Egypt

**Keywords:** Macrophage, TAM, MPNs, CD68, CD163, JAKV617F, ET, PMF, PV

## Abstract

**Background:**

Myeloproliferative neoplasms (MPNs) are clonal hematopoietic disorders with variable clinical outcomes influenced by the bone marrow microenvironment. Tumor-associated macrophages (TAMs), particularly the M1 (CD68⁺) and M2 (CD163⁺) subtypes, play critical roles in inflammation, fibrosis, and immune modulation. This study evaluates CD68- and CD163-positive macrophage frequencies across MPN subtypes and their clinical/prognostic significance.

**Methods:**

In this retrospective cohort study, 121 patients with histopathologically confirmed BCR::ABL1-negative, JAK2V617F-positive MPNs were assessed for CD68 and CD163 expression. The CD68/CD163 ratio was analyzed for associations with thrombosis, leukemic/fibrotic transformation, and survival outcomes using receiver operating characteristic (ROC) curves and Kaplan-Meier analyses.

**Results:**

A CD68/CD163 ratio > 1.63 correlated with shorter thrombosis-free survival (41.4 vs. 68.2 months; *p* = 0.001; hazard ratio [HR] 2.45, 95% confidence interval [CI] 1.45–4.14) and secondary myelofibrosis progression-free survival (48.3 vs. 79.0 months; *p* = 0.001; HR 2.67, 95% CI 1.55–4.60). The ratio predicted thrombosis (area under the curve [AUC] = 0.677, 95% CI 0.58–0.77; *p* = 0.001) and secondary myelofibrosis (AUC = 0.779, 95% CI 0.69–0.87; *p* < 0.001). CD68 alone showed excellent diagnostic accuracy for the prediction of secondary myelofibrosis (AUC = 0.851, 95% CI 0.78–0.92; specificity = 100%).

**Conclusions:**

TAM polarization, reflected by the CD68/CD163 ratio, is a prognostic marker in MPNs, particularly for thrombosis and fibrotic progression. These findings support integrating TAM profiling into routine histopathology and suggest macrophage-targeted therapies as potential strategies for MPN management.

**Supplementary Information:**

The online version contains supplementary material available at 10.1186/s13000-025-01727-x.

## Introduction

 Myeloproliferative neoplasms, namely the BCR::ABL1-negative subtypes such as polycythemia vera (PV), essential thrombocythemia (ET), and primary myelofibrosis (PMF), are identified as clonal hematopoietic stem cell conditions marked by the aberrant proliferation of myeloid lineages. Over the past two decades, the reported frequency of these neoplasms has elevated, likely due to enhanced diagnostic techniques and greater clinical awareness. As therapeutic approaches have evolved, especially with the introduction of JAK inhibitors and improved supportive care, the overall survival (OS) of patients with MPNs has shown marked improvement [1]. Despite these advancements, the clinical course of MPNs remains variable, with some patients progressing to advanced fibrosis or leukemic transformation. Increasing attention has been given to the role of the bone marrow microenvironment in MPN progression, particularly the involvement of tumor-associated macrophages. These macrophages have a critical role in inflammation, fibrosis, and immune modulation. Specifically, they contribute to the differentiation of mesenchymal stromal cells into myofibroblasts, thereby promoting marrow fibrosis, which is an indicator of advanced disease and poor prognosis [2, 3]. Moreover, tumor-associated macrophages (TAMs) exhibit functional plasticity, polarizing toward either a pro-inflammatory, antitumor phenotype (M1, CD68-positive) or an anti-inflammatory, tumor-promoting phenotype (M2, CD163-positive). A predominance of M2 macrophages has been associated with enhanced fibrosis, immunosuppression, and inferior outcomes in hematologic malignancies [4]. These findings position macrophages as potential targets for novel therapeutic strategies in MPNs.

This research aimed to assess the frequency of CD68- and CD163-positive macrophages across different subtypes of MPNs and to investigate their association with clinical and pathological features. By characterizing the pattern of TAM infiltration, we sought to elucidate its prognostic value and explore its potential as a therapeutic target in MPN management.

## Materials and methods

### Ethical approval

The study methodology received endorsement from the institutional research and ethics council of Kasr Al-Ainy Cairo University (Code N-34–2025).

### Patients and study design

In this retrospective cohort, a total of one hundred and twenty-one patients with histopathologically proven MPNs evaluated for the macrophage markers expression (CD163 and CD68) in trephine BMB attended the Hematology Department, Kasr A-Ainy School, Cairo University, during the period between January 2019 and January 2024.

### Sample size

A minimum sample size of 106 patients diagnosed with JAK2-Positive MPN was required to identify an effect size of 0.5 regarding the correlation between CD68/CD163 ratio and thrombosis and progression to secondary fibrosis, with a significance level of 0.05 and 85% power in a one-tailed test.

### Inclusion criteria

available paraffin blocks and full clinical data; denovo patient with JAK2V617 positive PV, ET, PMF, and MPN-U with regard to the 5th edition of WHO hematolymphoid malignancies criteria; males and females; and Egyptians.

### Exclusion criteria

non-Egyptians or referred materials with unavailable paraffin blocks or full clinical data.

**Fig. 1:**
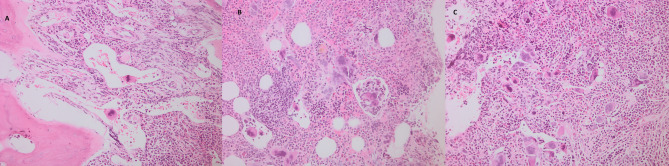
Histopathology of a fibrotic-phase primary myelofibrosis case. (A–C) Megakaryocyte atypia with clustering, dilated marrow sinusoids, and intrasinusoidal megakaryopoiesis. Hematoxylin and eosin stain, original magnification ×100

**Fig. 2:**
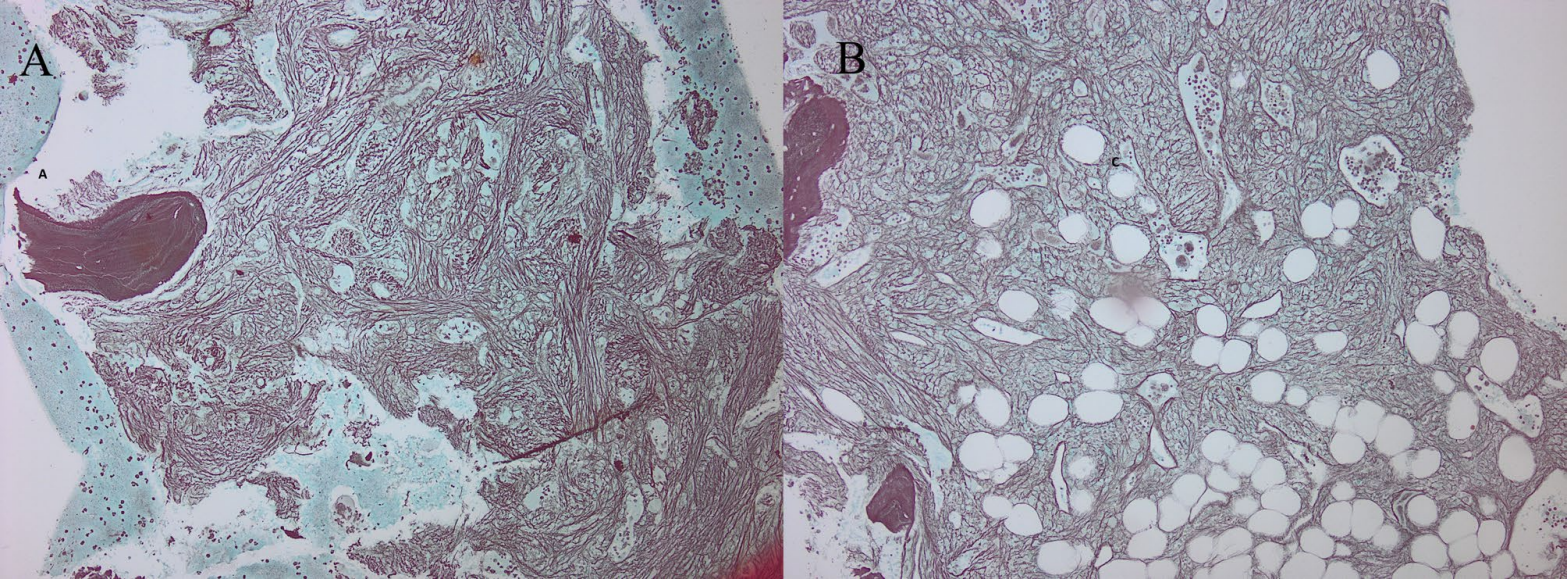
Reticulin staining in primary myelofibrosis. (A, B) Coarse intersecting bundles of black-stained fibers. Reticulin silver stain, original magnification ×100

**Fig. 3:**
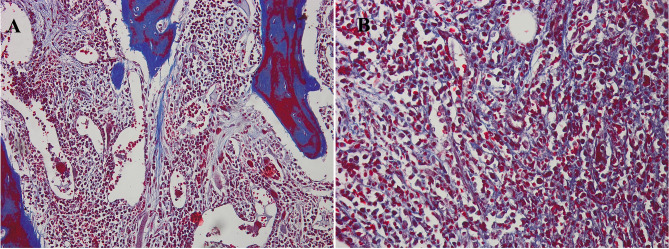
Collagen staining in primary myelofibrosis. (A) Coarse blue-stained fibers, original magnification ×100; (B) higher magnification, ×400. Masson’s trichrome stain

The hematoxylin- and eosin-stained, silver-stained reticulin, and trichrome slides of the selected cases were reviewed for histopathologic data, including the histologic kind of neoplasm, reticulin and collagen fibrosis grade. For illustrative purposes, representative photomicrographs of bone marrow sections from a fibrotic-phase primary myelofibrosis case are shown in Figs. 1,2 and 3. Data regarding gender, age, hemoglobin (Hb), total leucocytic count (TLC), peripheral blood (Pb) blast count, bone marrow aspirate (BMA) blast count, thrombosis, JAK2V617F mutation allele burden, OS, secondary fibrosis Progression-Free Survival (PFS), Thrombosis-Free Survival (TFS) and Leukemia-Free Survival (LFS) have been gathered from the case’s clinical files and monitoring information. The OS was defined as the time from MPN diagnosis to death from any cause or last follow-up. The TFS was the time from diagnosis to the occurrence of the first unprovoked thrombosis. The PFS was the interval from diagnosis to the development of secondary myelofibrosis. The LFS was the time to evolution to acute leukemia. Thrombosis events were systematically assessed using standardized protocols: (1) Baseline evaluation: Comprehensive medical history, physical examination, and coagulation studies. (2) Regular monitoring: Clinical assessment and D-dimer measurement every 3–6 months. (3) Event confirmation: Imaging studies when clinically indicated (Doppler ultrasonography for venous thrombosis, CT angiography for arterial events). (4) Inclusion criteria: Only objectively confirmed, unprovoked thrombotic events occurring ≥ 30 days post-MPN diagnosis. (5) Adjudication: All events were reviewed by two independent doctors using standardized diagnostic criteria. Systematic viral screening included: HBsAg and anti-HBc for HBV; anti-HCV antibodies with confirmatory HCV RNA quantification when positive; and HIV screening via ELISA methodology.

### Immunohistochemistry analysis

Paraffin-embedded tissue sections with a three-micrometer thickness were prepared for immunohistochemistry (IHC) of macrophage markers utilizing CD163 and CD68 (Dako), following the manufacturer’s protocols. Slides were dried at sixty-five degrees Celsius, placed in retrieval solution (pH 6.0, Medac PMB-1–250), and washed utilizing washing buffer (Medac B1-30 A), followed by distilled water. Endogenous peroxidase activity was blocked utilizing H₂O₂. IHC was then conducted using primary antibodies against CD163 (clone MRQ-26, Medac, 1:1000) and CD68 (clone PGM1, Agilent, 1:100). Photomicrographs have been taken utilizing a BX51 microscope (Olympus, Germany) and a Zeiss AxioCam MRc5 camera in conjunction with Axiovision software (Carl Zeiss, Germany).

**Fig. 4:**
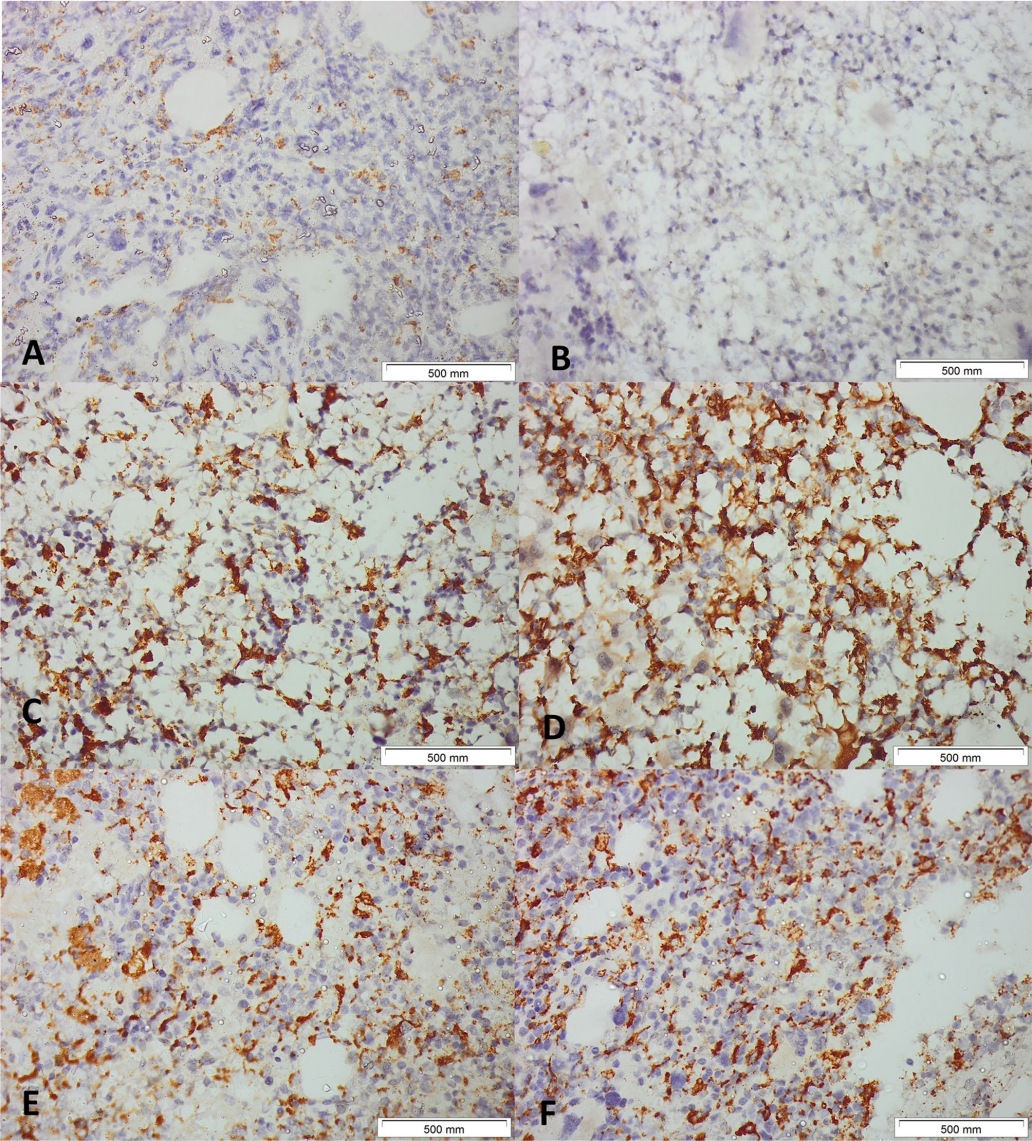
Immunohistochemical Detection of CD68+ Macrophages in JAK2-Positive Myeloproliferative Neoplasms (MPN). Representative images of bone marrow tissue sections from patients with JAK2-positive MPN subjected to CD68 immunohistochemistry. Panels (**A**–**F**) illustrate the distribution and intensity of CD68-positive macrophages across different cases. **A** and **B**: Samples demonstrating sparse CD68+ macrophages scattered throughout the marrow.** C** and **D**: Marked increase in CD68+ cells, with extensive clustering and network formation among adipocytic spaces. **E** and **F**: High-density infiltration by CD68+ macrophages in cellular marrow regions. Hematoxylin was used as a counterstain. Original magnification, ×400, Scale bars: 500μm

**Fig. 5:**
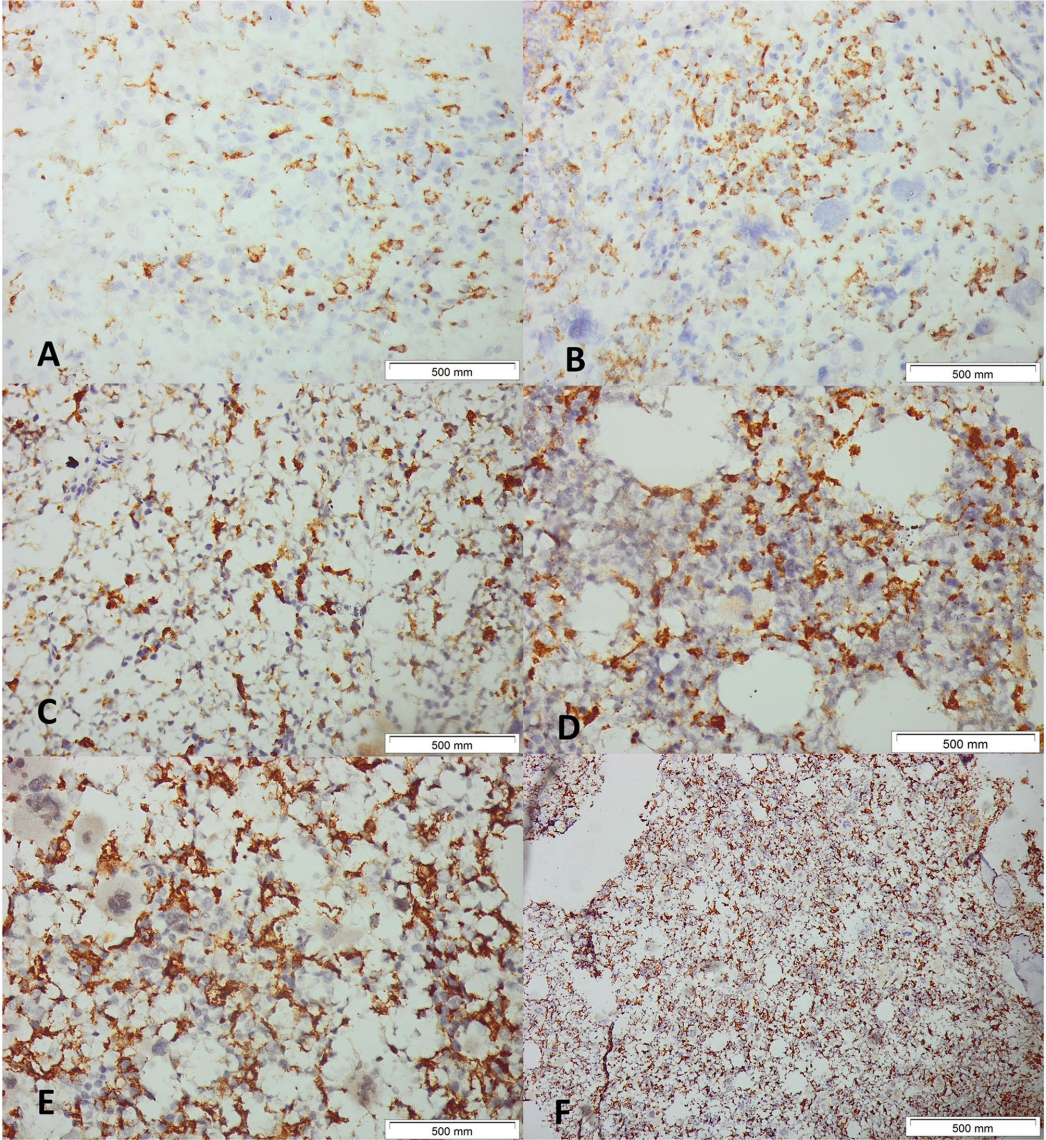
Immunohistochemical Detection of CD163+ Macrophages in JAK2-Positive Myeloproliferative Neoplasms (MPN). Representative images of bone marrow tissue sections from patients with JAK2-positive MPN subjected to CD163 immunohistochemistry. Panels (**A**–**F**) show the distribution and density of CD163-positive macrophages across different cases.**A** and **B**: Sparse CD163+ macrophages dispersed throughout the marrow. **C** and **D**: Moderate increase in CD163+ cells, with clustering and network formation among adipocytic spaces. **E** and **F**: Dense infiltration by CD163+ macrophages creating a sponge-like reticular network in highly cellular regions. CD163+ macrophages typically display a stellate morphology with slender cytoplasmic processes. Hematoxylin was used as a counterstain. Original magnification, ×400 for Panels**A**-**E**, x200 for Panel **F**, Scale bars: 500μm

CD68- or CD163-positive cells per all nucleated cells inside all assessable bone marrow regions—excluding hemorrhagic, bony, or crushed areas—were documented to guarantee that identical fields were analyzed by the observers. The quantity of CD163- and CD68-positive cells in the IHC sections was assessed by two independent observers (blinded to clinical data) analyzing the photographed fields (Figs. 4 and 5). CD68 + and CD163 + cells were counted manually in non-overlapping high-power fields, expressed as a percentage of total nucleated cells; discrepancies > 10% were reviewed jointly to reach consensus.

### Statistical analyses

This study used standard retrospective cohort data statistics. Continuous variables were medians and interquartile ranges, whereas categorical variables were frequencies and percentages. To compare groups, non-parametric tests like Mann–Whitney U and Kruskal–Wallis for continuous variables and chi-square or Fisher’s exact for categorical variables were used. Spearman’s or Pearson’s correlation analysis examined clinical, laboratory, and immunohistochemistry parameter correlation. Thrombosis predictive variables were found in univariate and multivariate Cox proportional hazards regression and logistic regression. AUC, sensitivity, and specificity were assessed using receiver operating characteristic (ROC) curves to define macrophage marker diagnostic cut-off values. Survival results were estimated and compared using Kaplan–Meier and log-rank tests.

## Results

In this retrospective cohort, a total of 121 MPN cases were included; their descriptive data is represented in Table 1. The observed secondary myelofibrosis rate among ET patients (32%) reflected rigorous case review. Cases were re-evaluated to exclude misclassification of pre-fibrotic myelofibrosis; both initial diagnostic and subsequent disease progression criteria followed WHO 5th edition guidelines, and independent pathologists re-examined clinical as well as pathological data. HCV infection status was carefully documented and analyzed due to its relatively high prevalence in Egypt. Moreover, underlying chronic liver disease may contribute to confounding laboratory and clinical features.

**Table 1:** Baseline characteristics, disease features and outcomes by JAK2-positive myeloproliferative neoplasms (MPN) subtype

		PV(*n* = 34)	ET(*n* = 25)	PMF(*n* = 54)	MPN-U(*n* = 8)
Age (years)		61 (55–65)	55 (49–60)	62(51–69)	65.5 (55–70)
Gender, n(%)	Male	16 (47.1)	8(32)	24(44.4)	1(12.5)
	Female	18 (52.9)	17(68)	30(55.6)	7(87.5)
Constitutional symptoms*, n(%)	Yes	3 (8.8)	0(0)	29(53.7)	6(75)
	No	31 (91.2)	25(100)	25(46.3)	2(25)
spleen size (cm)**		13(12–15)	12(11–13.5.5)	17.5(13–19)	18.5 (15.5–19.5)
Liver, n(%)	Cirrhosis	8 (23.5)	1(4)	16(29.6)	3(37.5)
	Hepatomegaly	5 (14.7)	0(0)	11(20.4)	1(12.5)
	Normal	21 (61.8)	24(96)	27(50)	4(50)
HBV, n(%)	Negative	34 (100)	25(100)	54 (100	8 (100)
HCV, n(%)	Positive	2 (5.9)	1(4)	15(27.8)	0(0)
	Negative	32 (94.1)	24(96	39(72.2)	8(100)
HIV, n(%)	Negative	34 (100)	25(100)	54(100)	8(100)
Subclassification of PMF, n(%)	Pre-fibrotic phase	-	-	16(29.6)	-
	Fibrotic phase	-	-	38(70.4)	-
DIPPS, n(%)	1	-	-	10 (18.5)	-
	2	-	-	10 (18.5)	-
	3	-	-	13(24.1)	-
	4	-	-	13(24.1)	-
	5	-	-	8(14.8)	-
IPSET, n(%)	Score 2	-	15(60)	18(33.3)	-
	Score 4	-	10(40)	36(66.7)	-
Hb (g/dl)		17.85(17–18.5.5)	12.30(10.5–13.7)	8(7–9.5.5)	8 (7.85- 10)
Hematocrit (%)		53.5 (51–55.4.4)	36.9 (31.5–41.1)	24 (21–28.8.8)	25.2 (23.8–30)
TLC (x10^3/cmm)		10.65(9–11.8.8)	7.20(6.6–8.6)	13.50(7.8–16)	15 (14.3–16.3)
Absolute neutrophilic count (x10^3/cmm)		7.562 (6.284–8.508)	5.112 (3.99–5.538)	9.31 (6.045–11.031)	10.177 (9.805–11.663)
Platelets (x10^3/cmm)		490(420–600)	850(775–920)	455(220–553)	515.5 (397–685.5.5)
Pb blasts (%)		0 (0–0)	0(0–0)	1(0–4)	0.5 (0–1.5.5)
LDH (mg/dl)		390(210–405)	198(177–340)	425.5(355–560)	435 (300–447)
JAK2 V617F%		39 (25.7–66)	50(40–75)	40(25–55)	24.5 (19–63.51.51)
BMA blast (%)		2 (1–4)	2(1–3)	4(2–5)	3.5(2.50–5.50)
CD34 positive cells (%)		0 (0–0)	0(0–0)	10(5–52)	5 (4.5–6.5)
Microvessel density, n(%)	Low	34 (100)	25(100)	23(42.6)	1(12.5)
	Moderate	0(0)	0(0)	25(46.3)	4(50)
	High	0(0)	0(0)	6(11.1)	3(37.5)
WHO Reticulin fibrosis grade, n(%)	0	20(58.8)	16(64)	0(0)	0(0)
	1	11(32.4)	8(32)	10(18.5)	1(12.5)
	2	1(2.9)	1(4)	14(25.9)	3(37.5)
	3	2(5.9)	0(0)	30(55.6)	4(50)
WHO Collagen grade, n(%)	0	31(91.2)	22(88)	12(22.2)	0(0)
	1	1(2.9)	3(12)	17(31.5)	6(75)
	2	2(5.9)	0(0)	12(22.2)	1(12.5)
	3	0(0)	0(0)	13(24.1)	1(12.5)
Unprovoked thrombosis after diagnosis, n(%)	Yes	7(20.6)	7(28)	21(38.9)	7(87.5)
	No	27(79.4)	18(72)	33(61.1)	1(12.5)
Type of thrombosis, n(%)	Arterial	0(0)	0(0)	2(9.5)	1(14.3)
	Venous	7(100)	7(100)	19(90.5)	6(85.7)
TFS (months)		40 (19–46)	23(17–40)	13(6–34)	7.5(5.5–8.5)
Acute leukemia transformation, n(%)	Yes	0(0)	0(0)	17(31.5)	0(0)
	No	34(100)	25(100)	37(68.5)	8(100)
LFS (months)		42 (39–52)	34(22–40)	26(11–40)	22.5 (13.5–40)
Progression to secondary myelofibrosis, n(%)	Yes	4(11.8)	8(32)	0(0)	0(0)
	No	30(88.2)	17(68)	0(0)	0(0)
PFS (months)		41.5 (33–52)	27(18–40	-	0 (0–0)
End of study status, n(%)	Alive	29(85.3)	25(100)	26(48.1)	7(87.5)
	Dead	5(14.7)	0(0)	28(51.9)	1(12.5)
Cause of death, n(%)	Leukemia	0(0)	0(0)	17(60.7)	0(0)
	Bleeding	2(40)	0(0)	3(10.7)	0(0)
	Sepsis	1(20)	0(0)	3(10.7)	0(0)
	Natural causes ✝	2(40)	0(0)	5(17.9)	1(100)
OS (months)		42 (39–52)	34(22–40	27(14–40)	22.5(13.5–40)

* Constitutional symptoms are defined as fever, night sweats, or weight loss > 10% in 6 months

** Spleen size was evaluated by abdominal ultrasound and recorded as the largest dimension in centimeters

✝ Natural causes: deaths deemed unrelated to MPN or its complications (e.g., age-related or cardiovascular event unrelated to MPN. Quantitative data presented as median (interquartile range), *PV* polycythemia rubra vera, *ET* essential thrombocytosis, *PMF* primary myelofibrosis, *MPN-U* myeloproliferative neoplasm unclassified, *DIPPS* Dynamic International Prognostic Scoring System, *IPSET* International Prognostic Score for Essential Thrombocytosis, *OS* overall survival, *LFS* Leukemia-Free Survival, *PFS* Secondary myelofibrosis Progression-Free Survival, *TFS* Thrombosis-Free Survival

### Association analysis

**Fig. 6:**
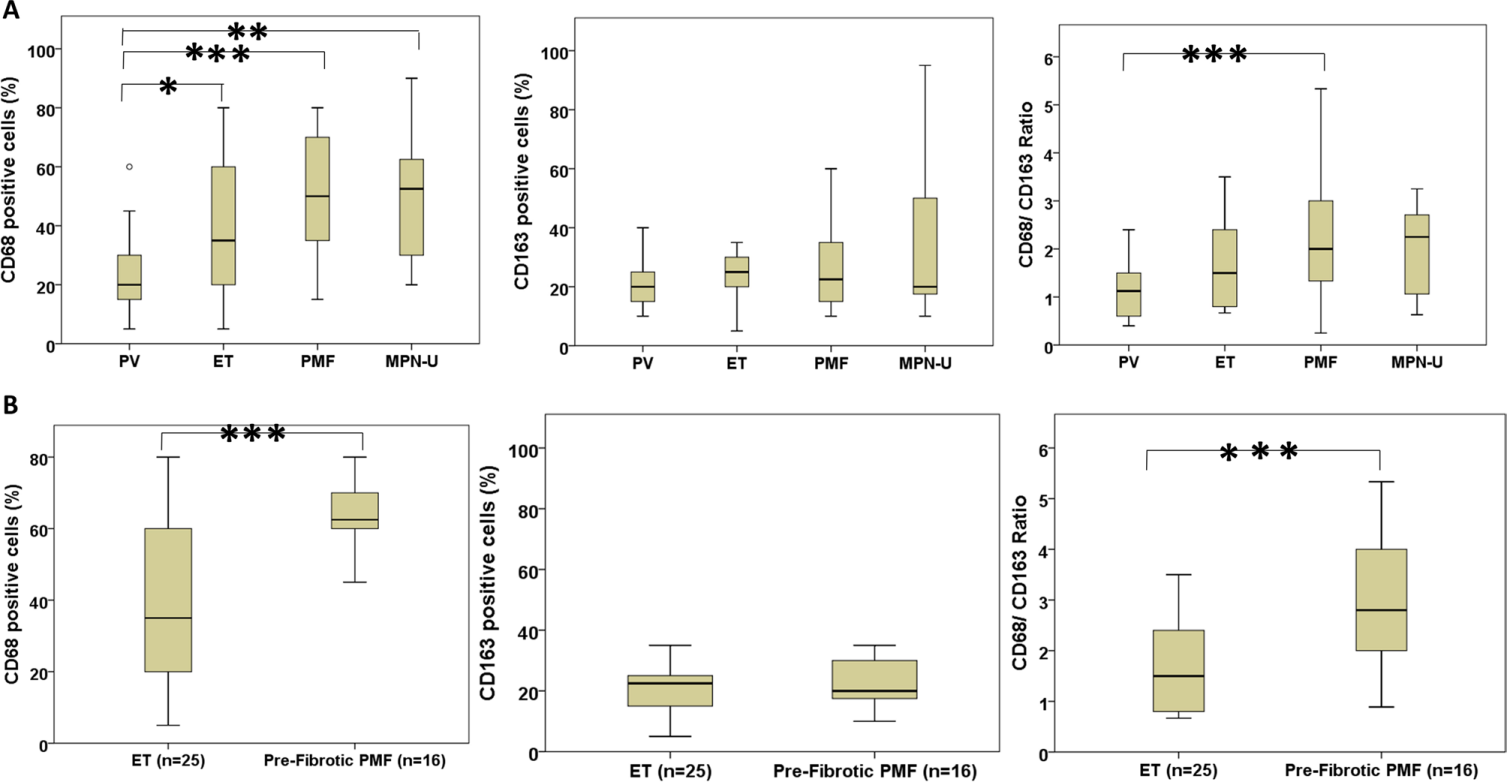
Bone marrow macrophage marker profiles distinguish myeloproliferative neoplasm (MPN) subtypes and disease stages. (**A**) Box plots show the percentages of CD68-positive cells (left), CD163-positive cells (middle), and the CD68/CD163 ratio (right) in bone marrow biopsies from patients with Polycythemia Vera (PV, *n*=34), Essential Thrombocythemia (ET, *n*=25), Primary myelofibrosis (PMF, *n*=54), and unclassifiable MPN (MPN-U, *n*=8). ET and PMF cases displayed significantly higher CD68-positive cell percentages and CD68/CD163 ratios compared to PV (Kruskal–Wallis test with post hoc tests; *p<0.05, **p<0.01, ***p<0.001). No significant differences in CD163 positivity were observed among groups.(**B**) Comparative analysis of ET (*n*=25) and pre-fibrotic PMF (*n*=16) for CD68-positive cells (left), CD163-positive cells (middle), and CD68/CD163 ratio (right). Pre-fibrotic PMF showed substantially increased CD68-positive cell percentages and CD68/CD163 ratios relative to ET (Mann–Whitney U test; ***p<0.001), while CD163 positivity remained simila

Association studies of CD68, CD163, and Their Ratio Across Diagnostic Groups are summarized in Table 2; Fig. 6.

**Table 2:** Association of immunohistochemical expression of CD68 and CD163 in myeloproliferative neoplasm subtypes

	PV(*n* = 34)	ET(*n* = 25)	PMF(*n* = 54)	MPN-U(*n* = 8)
CD68 positive cells (%)	20 (15–30)	35(20–60)^**a**^	50(35–70)^**c**^	52.5 (30–62.5.5)^**a**^
CD163 positive cells (%)	20 (15–25)	25(20–30)	22.50(15–35)	20 (17.5–50)
CD68/CD163 Ratio	1.13 (0.6–1.5)	1.5 (0.8–2.4)	2(1.33–3.33)^**c**^	2.25(1.06–2.71)

Data presented as median and interquartile range. ^a^*p*<0.05, ^b^*p*<0.01, ^c^*p*<0.001: PV vs. other groups. ^d^*p*<0.05, ^e^*p*<0.01, ^f^*p*<0.001: ET vs. other groups. ^g^*p*<0.05, ^h^*p*<0.01, ^j^*p*<0.001: PMF vs. other groups. ^g^*p*<0.05, ^h^*p*<0.01: MPN-U vs. other group

Abbreviations: *PV* polycythemia vera, *ET* essential thrombocythemia, *PMF* primary myelofibrosis, *MPN-U* myeloproliferative neoplasm unclassified

Data are presented as box plots indicating median, interquartile range (IQR), and full range. Statistical significance: *p < 0.05, **p < 0.01, ***p < 0.001. These findings indicate that elevated CD68 expression and CD68/CD163 ratio characterize ET and (pre-fibrotic) PMF, implicating shifts in macrophage polarization in MPN pathogenesis.

### Correlation analyses

**Fig. 7:**
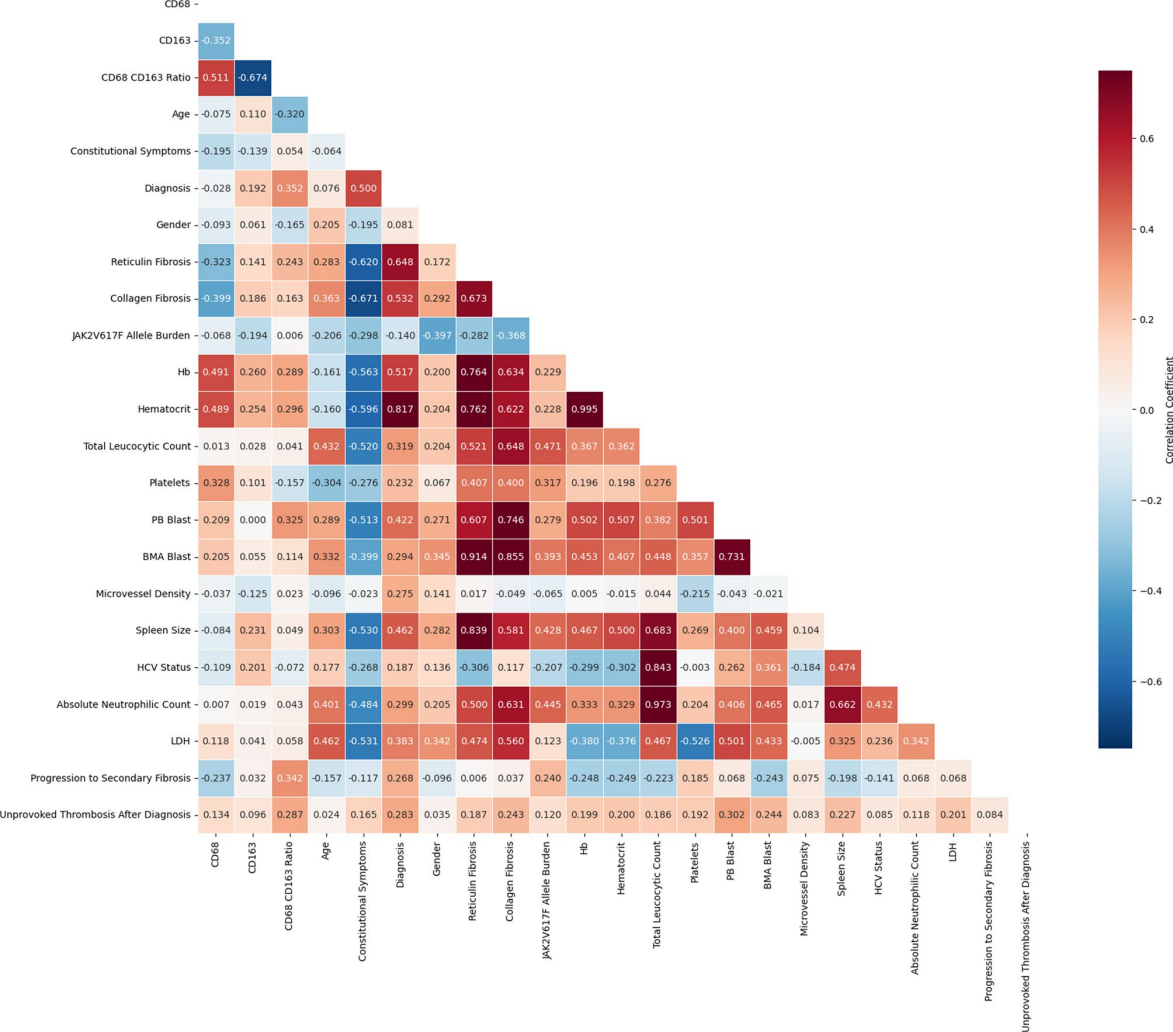
Heat map of correlations between CD68, CD163, and CD68/CD163 ratio with clinical and laboratory parameters in JAK2-positive myeloproliferative neoplasms

Correlation analysis between macrophage markers and clinicopathologic variables is summarized in Supplementary Tables 3 and Fig. 7. The heatmap demonstrates that higher CD68/CD163 ratios were found to correlate with markers of disease progression and adverse clinical events, such as increased risk of thrombosis and progression to secondary myelofibrosis Supplementary Table 3.

### Risk factors for thrombosis

The Cox proportional hazards regression analysis was conducted to identify baseline variables associated with the risk of thrombosis. Univariate analysis revealed that elevated LDH, higher BMA blast percentage, increased WHO collagen grade, and a higher CD68/CD163 ratio were significantly associated with increased thrombosis risk. The reduced multivariate model identified LDH, PMF diagnosis, CD68/CD163 ratio, and the ordered MPN diagnostic category as independent predictors of thrombosis. The model showed good predictive performance, with a concordance index of 0.761 (Table 3).

**Table 3:** Cox proportional hazards regression analysis for thrombosis (*n* = 42)

Variable	Univariate HR (95% CI)	Univariate *p*-value	Multivariate HR (95% CI)	Multivariate *p*-value
Age (years)	1.008 (0.978–1.038)	0.603	–	–
Male gender	1.204 (0.653–2.219)	0.552	–	–
JAK2 V617F allele burden (%)	0.990 (0.976–1.004)	0.148	–	–
LDH (mg/dL)	1.004 (1.002–1.006)	< 0.001	1.005 (1.002–1.007)	< 0.001
BMA blasts (%)	1.242 (1.083–1.424)	0.002	–	–
WHO collagen grade	1.505 (1.164–1.946)	0.002	–	–
PMF diagnosis	1.586 (0.863–2.913)	0.137	0.274 (0.120–0.627)	0.002
CD68/CD163 ratio	1.478 (1.169–1.870)	0.001	1.472 (1.115–1.944)	0.006
MPN diagnosis (ordered)	–	–	1.838 (1.278–2.643)	0.001

Model performance: Concordance index = 0.761

Univariate analysis was performed for each variable individually; multivariate analysis was performed using a reduced model including variables with p < 0.1 in univariate analysis and those of clinical significance

*HR* Hazard ratio, *CI* Confidence interval

* “–” *indicates variable was not included in the reduced multivariate model

Multivariate logistic regression analysis was performed to identify independent risk factors for thrombosis while controlling for potential confounding variables. Three variables emerged as statistically significant independent predictors of thrombosis risk (Table 4). Patients with higher CD68/CD163 ratios had approximately 68% increased odds of developing thrombosis compared to those with lower ratios. Additionally, each 1 mg/dl increase in lactate dehydrogenase (LDH) levels was associated with a 0.7% increase in the odds of thrombosis. Each cm increase in spleen size increases thrombosis risk by 37%. All three biomarkers maintained their statistical significance after adjusting for other covariates in the multivariate model.

**Table 4:** Multivariate logistic regression analysis for risk of thrombosis in myeloproliferative neoplasm patients

Variable*	Adjusted OR	95% CI	*P*-value
Age	0.942	(0.885–1.002)	0.057
Gender	0.471	(0.166–1.340)	0.158
Spleen size	1.370	(1.075–1.745)	0.011
HB	1.024	(0.835–1.256)	0.817
TLC	0.973	(0.825–1.147)	0.745
Platelets	1.000	(0.997–1.003)	0.907
Pb blasts	1.503	(0.957–2.361)	0.077
HCV	0.528	(0.098–2.832)	0.456
LDH	1.007	(1.002–1.012)	0.006
CD68	0.959	(0.913–1.007)	0.094
CD163	1.040	(0.992–1.090)	0.108
CD68/CD163 ratio	4.068	(1.486–11.140)	0.006
Reticulin grade	0.493	(0.224–1.085)	0.079
JAK2V617F allele burden	0.994	(0.970–1.019)	0.639

*Variables Excluded Due to Multicollinearity: Hematocrit (excluded, *r* = 0.999 with Hemoglobin), ANC (excluded, *r* = 0.971 with TLC), BMA blasts (excluded, *r* = 0.731 with Pb blasts) and Collagen grade (excluded, *r* = 0.825 with Reticulin grade)

Abbreviations: *TLC* total leukocyte count, *PB* peripheral blood, *LDH* lactate dehydrogenase, *HB* hemoglobin, *HCV* hepatitis C virus

To address potential HCV confounding, we performed sensitivity analyses excluding HCV-positive patients (*n* = 18). In the HCV-negative cohort (*n* = 103): CD68/CD163 ratio maintained significant predictive value for thrombosis (OR = 1.64, 95% CI:1.119–2.404, *p* = 0.011) in the HCV-negative cohort, confirming the robustness of our findings independent of viral hepatitis status. The effect size remained clinically meaningful despite the reduced sample size. HCV status was not a significant predictor in multivariate models (*p* = 0.423). Our HCV prevalence (14.9%) aligns with current Egyptian epidemiology following successful national treatment programs, confirming this is not an unusual finding [5, 6]. 

### Predictive performance: ROC curve analyses

**Fig. 8:**
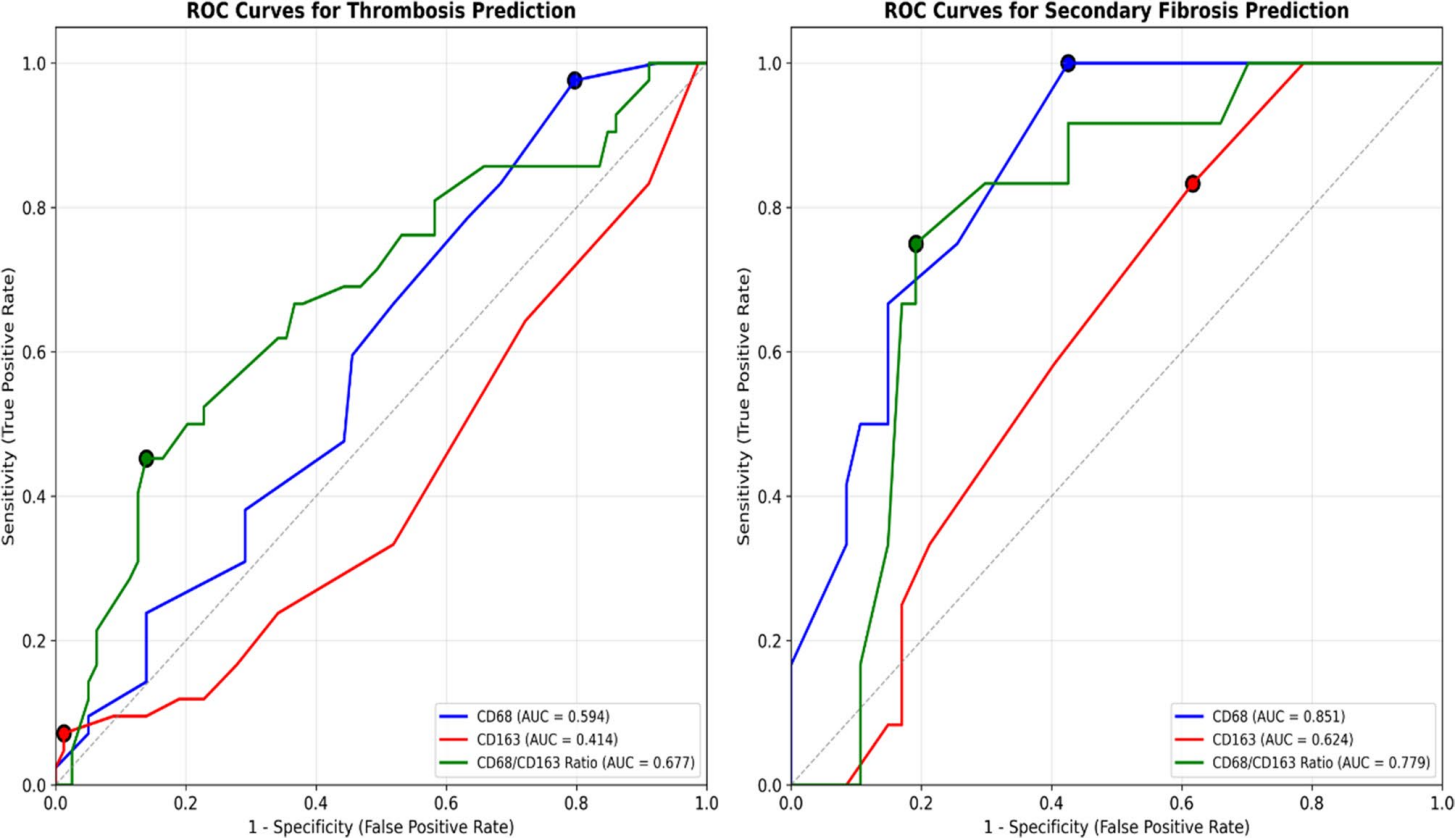
Receiver operating characteristic (ROC) curve analyses for CD68, CD163, and the ratio CD68/CD163 for the prediction of thrombosis and secondary myelofibrosis. In both panels, the ratio CD68/CD163 has more discriminative power than CD68 and CD163 individually

Diagnostic Performance of Macrophage Markers for Thrombosis and Secondary Myelofibrosis.

is presented in Supplementary Tables 6 and Fig. 8. ROC curve analyses demonstrated that the CD68/CD163 ratio provided the best diagnostic accuracy for both thrombosis and secondary myelofibrosis, outperforming individual markers. Using a cut-off of 1.63, the CD68/CD163 ratio yielded a sensitivity of 66.7% and specificity of 63.3% for thrombosis prediction (AUC = 0.677), and for secondary myelofibrosis, a cut-off of 1.55 gave a sensitivity of 80.9% and specificity of 75% (AUC = 0.779).

### Survival analyses

**Fig. 9:**
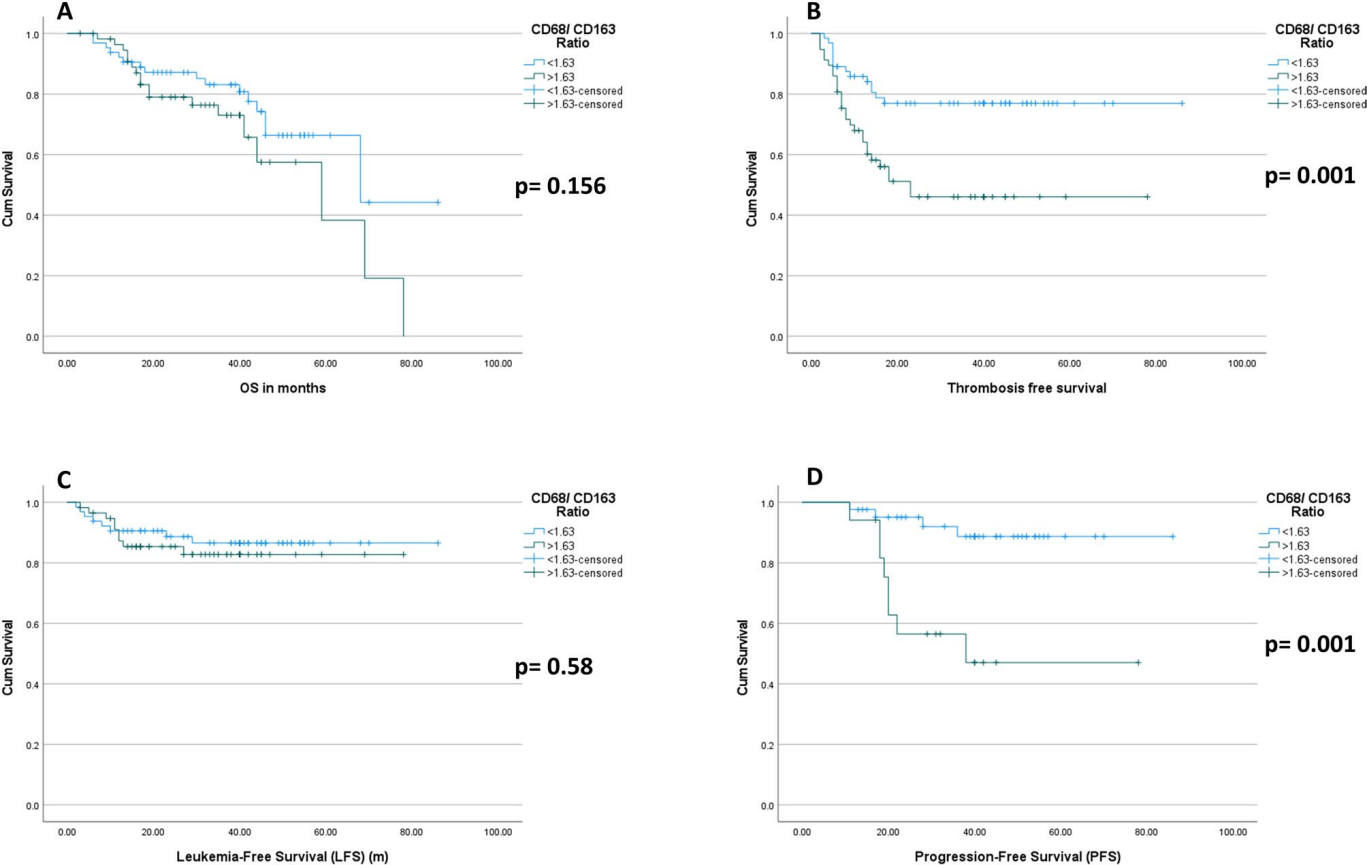
Prognostic significance of the CD68/CD163 ratio in JAK2-Positive Myeloproliferative Neoplasms: Kaplan–Meier curves display cumulative survival probabilities for patients stratified by the CD68/CD163 macrophage marker ratio (≤1.63 vs.>1.63), highlighting its prognostic value across multiple clinical outcomes. The cutoff value of 1.63 was determined by ROC analysis

Patients with a CD68/CD163 ratio < 1.63 had longer OS compared to those with a ratio > 1.63, but the variance was statistically insignificant. A CD68/CD163 ratio > 1.63 was significantly associated with shorter TFFS and secondary myelofibrosis PFS. The mean LFS was longer in patients with a CD68/CD163 ratio < 1.63 compared to those with a ratio > 1.63. However, the difference was statistically insignificant, indicating that the CD68/CD163 ratio does not significantly affect LFS (Supplementary Table 7, Fig. 9).

(A) Overall survival (OS) shows no significant difference between groups (p = 0.156).
(B) Patients with a CD68/CD163 ratio > 1.63 exhibit reduced thrombosis-free survival (TFS) compared to those with a ratio ≤ 1.63 (p = 0.001).
(C) Leukemia-free survival (LFS) is not significantly influenced by the CD68/CD163 ratio (p = 0.58).
(D) Progression-free survival (PFS) is significantly shorter in patients with a ratio > 1.63 (p = 0.001).

## Discussion

TAMs, frequently derived from myeloid-derived suppressor cells, are integral components of the tumor microenvironment and are implicated in tumor initiation, progression, immune evasion, and therapy resistance. High TAM infiltration has been consistently related to poor results in several malignancies, including hepatocellular carcinoma, breast and colon tumors, and gliomas [2, 7]. However, our findings reveal a complex and paradoxical pattern in JAK2-positive MPNs where M1-shifted macrophages, rather than the traditionally implicated M2 phenotype, are associated with adverse clinical outcomes.

### The macrophage polarization paradox in JAK2-positive MPNs

To the best of our knowledge, scarce studies have examined macrophages in myeloproliferative neoplasms [8]. TAMs exhibit functional plasticity, polarizing toward either a pro-inflammatory, antitumor phenotype (M1, CD68-positive) or an anti-inflammatory, tumor-promoting phenotype (M2, CD163-positive). Conventionally, a predominance of M2 macrophages has been associated with enhanced fibrosis, immunosuppression, and inferior outcomes in hematologic malignancies [4, 9–24]. Interestingly, our findings reveal an apparent paradox where M1-shifted macrophages (higher CD68/CD163 ratio) are associated with adverse outcomes in JAK2-positive MPNs, contrasting with the conventional paradigm observed in solid tumors and other hematological malignancies where M2 macrophages typically drive pathology. This M1-associated pathology likely reflects the unique inflammatory microenvironment of MPNs, where chronic JAK2-driven inflammatory signaling creates a distinct macrophage activation pattern. The JAK2V617F mutation constitutively activates downstream pathways including STAT3 and NF-κB, creating a persistently inflamed bone marrow microenvironment that may skew macrophage activation toward sustained M1 polarization [8, 25]. This chronic inflammatory state establishes a feed-forward loop between mutated hematopoietic cells and tissue macrophages, resulting in pathological consequences of M1 activation that differ fundamentally from the typical M2-driven pathology observed in other cancers.

### Mechanistic basis for M1-associated thrombotic risk

To investigate the role of TAMs in thrombotic complications, ROC curve analysis was conducted. CD68 and CD163 alone demonstrated limited discriminatory power, with AUCs of 0.594 (*p* = 0.070) and 0.414 (*p* = 0.114), respectively. However, the CD68/CD163 ratio showed superior predictive performance, with an AUC of 0.677 (*p* = 0.001). At a cutoff value of 1.633, the ratio achieved a sensitivity of 66.7% and a specificity of 63.3%, suggesting that a higher ratio, reflecting a dominance of pro-inflammatory M1 macrophages, correlates with increased thrombotic risk. The association between M1-shifted macrophages and thrombotic risk in our cohort suggests several mechanistically plausible pathways. First, M1 macrophages are prolific producers of pro-inflammatory cytokines including IL-1β, TNF-α, and IL-6, which can activate endothelial cells, promote tissue factor expression, enhance platelet activation and aggregation, and stimulate hepatic synthesis of acute-phase proteins including fibrinogen and coagulation factors [26, 27]. Second, M1 macrophages directly express tissue factor, the primary initiator of the extrinsic coagulation cascade, providing a direct prothrombotic stimulus [28, 29]. Furthermore, M1-derived inflammatory mediators can promote neutrophil extracellular trap (NET) formation, which contributes significantly to thrombosis in MPNs through direct procoagulant effects and platelet activation [30, 31]. Recent studies have demonstrated that JAK2V617F-positive neutrophils exhibit increased spontaneous NET formation, and that M1 macrophage-derived cytokines can further enhance this prothrombotic mechanism [30, 32]. Additionally, sustained M1 activation may compromise endothelial integrity and anticoagulant function, creating a prothrombotic endothelial phenotype [33]. These mechanistic insights suggest that the CD68/CD163 ratio could serve as a clinically meaningful biomarker for thrombotic risk stratification, potentially guiding prophylactic anticoagulation strategies in high-risk MPN patients. The 88% increased thrombotic risk (HR = 1.88, 95% CI: 1.02–3.48) associated with higher ratios provides clinically significant risk stratification that could inform therapeutic decision-making.

### Fibrotic progression and M1 macrophages

Regarding fibrotic progression, CD68 alone demonstrated excellent diagnostic accuracy for secondary myelofibrosis prediction, with an AUC of 0.851 (*p* < 0.001), achieving a specificity of 100% at a cutoff of less than 27.5. The CD68/CD163 ratio was also predictive of fibrotic transformation (AUC = 0.779, *p* < 0.001), with an optimal cutoff of less than 1.55 producing a sensitivity of 80.9% and specificity of 75%. The association between M1-shifted macrophages and fibrotic progression, while initially counterintuitive, may involve several mechanisms specific to the MPN microenvironment. Under the chronic inflammatory conditions created by JAK2V617F signaling, M1 macrophages may produce TGF-β and PDGF, promoting stromal cell proliferation and collagen deposition [34, 35]. Additionally, M1 macrophages may interact directly with megakaryocytes and fibroblasts within the altered MPN bone marrow niche, contributing to the complex cellular interactions that drive fibrotic transformation [3]. In MPNs, Molitor and colleagues showed increased macrophage frequency in PMF compared to PV (CD68: *p* < 0.001; CD163: *p* < 0.001) and ET (CD68: *p* < 0.001; CD163: *p* < 0.001), highlighting that macrophages played a key role in marrow fibrosis development by promoting mesenchymal stromal cell differentiation into profibrotic myofibroblasts [2]. Mechanistic studies have linked macrophage subsets to transcriptional programs and MPN subtype-specific pathologies, with CD163 correlating positively with hemoglobin in PV, platelets in ET, and negatively with hemoglobin in PMF [8]. 

### Prognostic implications and survival outcomes

Survival analyses further supported the prognostic significance of macrophage polarization in our JAK2-positive cohort. Patients with a CD68/CD163 ratio below 1.63 exhibited longer mean PFS (79.02 months versus 48.27 months, *p* = 0.001) and TFS (68.2 months versus 41.4 months, *p* = 0.001) compared with those with a ratio above 1.63. Although the difference in OS (63.2 versus 51.1 months) did not reach statistical significance (*p* = 0.156), a favorable trend was observed. The LFS was comparable between the groups (76 versus 67 months, *p* = 0.580), suggesting that the CD68/CD163 ratio may not be a strong predictor of leukemic transformation but remains highly relevant for disease control and thrombotic risk assessment.

### Contextualizing findings within hematological malignancies

While our findings contrast with studies in other hematological malignancies where M2 macrophages drive adverse outcomes, they align with emerging evidence suggesting disease-specific macrophage roles in myeloid malignancies. In adult T-cell leukemia/lymphoma, CD163-positive TAMs have been associated with poor prognosis [9]. In acute myeloid leukemia, high CD163 expression has been linked to shorter survival, whereas CD68 showed no prognostic correlation [10]. Gene expression profiling in large AML cohorts has demonstrated that increased expression of CD163, CD68, and CD206 is related to inferior OS [11, 12]. However, these studies predominantly focused on acute leukemias and lymphomas, which represent fundamentally different pathophysiological contexts from chronic MPNs. The chronic inflammatory state driven by JAK2V617F creates a unique microenvironment where persistent cytokine signaling may result in distinct macrophage functional consequences compared to acute malignancies. In multiple myeloma (MM), high infiltration of CD163-positive TAMs has been significantly associated with poorer outcomes and lower complete response rates, with CD163 confirmed as an independent prognostic factor [13–17]. Similarly, in Hodgkin lymphoma, cases with the highest M2 TAM counts exhibit significantly diminished survival outcomes [18–21]. In non-Hodgkin lymphoma, while CD68-positive content has been correlated with improved survival, an increased CD163/CD68 ratio predicts worse prognosis [23, 24]. 

### Disease-specific macrophage biology in MPNs

Our findings underscore the critical importance of understanding macrophage biology within the specific context of JAK2-positive MPNs. The JAK2V617F mutation creates a unique inflammatory milieu characterized by:

1. *Constitutive cytokine production*: JAK2V617F directly activates inflammatory pathways including STAT3 and NF-κB signaling, leading to sustained production of inflammatory mediators [25, 36].
2. *Altered hematopoietic-stromal interactions*: Mutated hematopoietic cells interact aberrantly with bone marrow stromal elements, including macrophages, creating feedback loops that perpetuate inflammation [37].
3. *Distinct clinical phenotypes*: Unlike acute leukemias, MPNs are characterized by thrombotic complications and progressive fibrosis, clinical features that may be specifically promoted by chronic M1 activation rather than M2-mediated immunosuppression.

### Therapeutic implications

The heterogeneity of TAMs in different malignancies underscores the importance of marker selection and standardization in TAM profiling. While M2-targeted therapeutic strategies may be appropriate for acute leukemias, lymphomas, and MM where M2 macrophages drive pathology, our findings suggest that therapeutic approaches targeting the inflammatory axis may be more relevant in JAK2-positive MPNs [4]. JAK2 inhibitors such as ruxolitinib may exert beneficial effects partly through modulating macrophage activation and reducing the chronic inflammatory state that drives M1-associated pathology in MPNs [38]. Additionally, anti-inflammatory approaches targeting IL-1β, TNF-α, or complement activation may represent novel therapeutic strategies for preventing thrombotic complications and fibrotic progression in high-risk patients identified through CD68/CD163 ratio assessment [37]. 

### Clinical integration and risk stratification

These findings highlight the clinical utility of TAM profiling, particularly the CD68/CD163 ratio, as a potential biomarker for identifying patients at risk of thrombotic complications and fibrotic progression in JAK2-positive MPNs. The superior predictive performance of the ratio compared to individual markers suggests that the balance between M1 and M2 macrophages, rather than absolute numbers, is the critical determinant of clinical outcomes.

Multivariate Cox proportional hazards regression modeling determined that both MPN subtype and CD68/CD163 macrophage polarization ratio were independent predictors of thrombosis timing in our cohort, supporting the clinical relevance of incorporating this biomarker into routine risk assessment algorithms.

### Limitations

We specifically focused on JAK2V617F-positive cases to ensure molecular homogeneity and to investigate the relationship between this driver mutation and macrophage polarization. The JAK2V617F mutation directly activates inflammatory pathways, including STAT3 and NF-κB signaling, making it an ideal model for exploring TAMs dynamics. While this approach limits the applicability of our findings to JAK2-mutated MPNs, it provides clearer mechanistic insight into mutation-specific microenvironmental interactions.

Our study has several additional limitations. First, it was retrospective and conducted at a single center. Second, molecular profiling was limited, and other acquired mutations—such as TET2, DNMT3A, and ASXL1—were not assessed. Although the total sample size of 121 patients exceeded the calculated minimum required to detect clinically meaningful effects, certain subgroups—particularly MPN-U (*n* = 8)—were small, which may have reduced statistical power and limits the generalizability of subtype-specific results.

Methodologically, because immunohistochemical analysis was performed on single-marker stained sequential sections rather than using double or multiplex IHC, we could not directly confirm single-cell co-expression or spatial co-localization of CD68 and CD163. We recommend that future studies incorporate multiplex staining approaches to address this limitation. Another limitation is that functional characterization of macrophage polarization was not conducted, as M1-associated cytokines (e.g., IL-1β, TNF-α) were not measured in this study, restricting deeper mechanistic insight.

Finally, the apparent contradiction between the M1-associated adverse outcomes observed in our cohort and the established M2-driven pathology in other cancers warrants further investigation. Validation in larger, multi-center cohorts and mechanistic studies is needed to elucidate the underlying pathways. In addition, future research should include CALR**-** and MPL-mutated cases to determine whether macrophage polarization patterns are consistent across all MPN driver mutations or are mutation-specific—an insight that could have important implications for personalized therapeutic strategies.

## Conclusion

Our findings highlight the complex and context-dependent role of macrophage polarization in JAK2-positive MPNs, where M1-shifted macrophages paradoxically contribute to adverse outcomes including thrombotic complications and fibrotic progression. The CD68/CD163 ratio emerged as a promising biomarker for assessing thrombotic risk and fibrotic progression, demonstrating superior predictive performance compared to individual markers. These results underscore the need for disease-specific understanding of tumor-associated macrophages and suggest that therapeutic strategies targeting the inflammatory axis, rather than conventional M2-targeted approaches, may be more relevant in JAK2-positive MPNs. These findings reinforce the potential value of incorporating TAM markers into routine histopathological assessment and highlight the need for further studies exploring macrophage-targeted therapeutic strategies specifically designed for the unique pathophysiology of MPNs.

## Supplementary Information

Supplementary Material 1: Supplementary Table 7. Survival Outcomes According to CD68/CD163 Ratio in JAK2-Positive Myeloproliferative Neoplasm Patients

Supplementary Material 2: Supplementary Table 6. Diagnostic Performance of Macrophage Markers for Thrombosis and Secondary Myelofibrosis.

Supplementary Material 3: Table 3: Correlation between Clinico-Laboratory parameters and macrophage marker expression (CD68 and CD163) in patients with JAK2-positive myeloproliferative neoplasms 

## Data Availability

The datasets generated and/or analyzed during the current study are available from the corresponding author upon reasonable request.

## Abbreviations

MPN: Myeloproliferative Neoplasm
TAM: Tumor-associated Macrophage
PV: Polycythemia Vera
ET: Essential Thrombocythemia
PMF: Primary Myelofibrosis
MPN-U: Myeloproliferative Neoplasm Unclassified
OS: Overall Survival
PFS: Progression-free Survival
TFS: Thrombosis-free Survival
LFS: Leukemia-free Survival
IHC: Immunohistochemistry
ROC: Receiver Operating Characteristic
AUC: Area Under the Curve
HR: Hazard Ratio
CI: Confidence Interval
