# Supplementary material for: Prognostic utility of macrophage polarization (CD68/CD163 ratio) in Egyptian JAK2 positive myeloproliferative neoplasm patients: a single center study
Source: Diagn Pathol. 2025 Nov 14;20:129. doi: 10.1186/s13000-025-01727-x (PMC12619198; doi:10.1186/s13000-025-01727-x)
Supplement: Supplementary file 1 — Supplementary Material 1: Supplementary Table 7. Survival Outcomes According to CD68/CD163 Ratio in JAK2-Positive Myeloproliferative Neoplasm Patients [file 13000_2025_1727_MOESM1_ESM.docx]

Table 7. Survival Outcomes According to CD68/CD163 Ratio in JAK2-Positive Myeloproliferative Neoplasm Patients

| Survival Parameter | CD68/CD163 Ratio | Mean (months) | Median (months) | 95% CI (months) | p-value |
| --- | --- | --- | --- | --- | --- |
| OS | <1.63 | 63.19 | 68 | 31.3 – 104.6 | 0.156 |
|  | >1.63 | 51.14 | 59 | 32.4 – 85.5 |  |
| TFS | <1.63 | 68.24 | NR | - | 0.001 |
|  | >1.63 | 41.36 | 23 | - |  |
| LFS | <1.63 | 75.99 | NR | - | 0.58 |
|  | >1.63 | 66.66 | NR | - |  |
| PFS | <1.63 | 79.02 | NR | - | 0.001 |
|  | >1.63 | 48.27 | 38 | - |  |

Abbreviations: OS: Overall Survival, TFS: Thrombosis-Free Survival LFS: Leukemia-Free Survival, PFS: Progression-Free Survival, CI, confidence interval; NR, not reached.

**Notes:**

- Survival estimates are reported as mean and median (months) with 95% confidence intervals where available.
- CD68/CD163 ratio cutoff: 1.63 (as determined by ROC analysis).
- P-values calculated by the log-rank test.
