# Supplementary material for: Prognostic utility of macrophage polarization (CD68/CD163 ratio) in Egyptian JAK2 positive myeloproliferative neoplasm patients: a single center study
Source: Diagn Pathol. 2025 Nov 14;20:129. doi: 10.1186/s13000-025-01727-x (PMC12619198; doi:10.1186/s13000-025-01727-x)
Supplement: Supplementary file 2 — Supplementary Material 2: Supplementary Table 6. Diagnostic Performance of Macrophage Markers for Thrombosis and Secondary Myelofibrosis. [file 13000_2025_1727_MOESM2_ESM.docx]

Table 6. **Diagnostic Performance of Macrophage Markers for Thrombosis and Secondary Myelofibrosis**

|  |  | **AUC** | **P value** | | **95% CI** | | | **Cut off** | | **Sensitivity %** | **Specificity %** |
| --- | --- | --- | --- | --- | --- | --- | --- | --- | --- | --- | --- |
| Thrombosis  (n=42) | **CD68 positive cells %** | 0.594 | 0.070 | | 0.492 | | 0.696 | - | | - | - |
|  | **CD163 positive cells %** | 0.414 | 0.114 | | 0.306 | | 0.521 | - | | - | - |
|  | **CD68/ CD163 Ratio** | 0.677 | 0.001 | | 0.574 | | 0.780 | 1.633 | | 66.7 | 63.3 |
| Secondary myelofibrosis  (n=12) | **CD68 positive cells %** | 0.851 | < 0.001 | 0.750 | | 0.952 | | < 27.5 | 57.4 | | 100 |
|  | **CD163 positive cells %** | 0.624 | 0.112 | 0.471 | | 0.777 | | - | - | | - |
|  | **CD68/ CD163 Ratio** | 0.779 | < 0.001 | 0.649 | | 0.909 | | < 1.55 | 80.9 | | 75 |

**Abbreviations:** AUC, area under the curve; CI, confidence interval

**Notes:**

- Sensitivity and specificity are shown for optimal cut-off (based on Youden’s index).
- “-” indicates no statistically significant cut-off was identified.
