# Supplementary material for: Prognostic utility of macrophage polarization (CD68/CD163 ratio) in Egyptian JAK2 positive myeloproliferative neoplasm patients: a single center study
Source: Diagn Pathol. 2025 Nov 14;20:129. doi: 10.1186/s13000-025-01727-x (PMC12619198; doi:10.1186/s13000-025-01727-x)
Supplement: Supplementary file 3 — Supplementary Material 3: Table 3: Correlation between Clinico-Laboratory parameters and macrophage marker expression (CD68 and CD163) in patients with JAK2-positive myeloproliferative neoplasms [file 13000_2025_1727_MOESM3_ESM.docx]

Table 3. Correlation Between Clinico-Laboratory Parameters and Macrophage Marker Expression (CD68 and CD163) in Patients With JAK2-positive Myeloproliferative Neoplasms

| Clinical and Laboratory Parameter | CD68⁺ Cells (%) r (p-value) | CD163⁺ Cells (%) r (p-value) | CD68/CD163 Ratio r (p-value) |
| --- | --- | --- | --- |
| Age | 0.27** | -0.13 | 0.34*** |
| Gender | -0.0930 | 0.0609 | -0.1652 |
| Spleen size (cm) | 0.19* | 0.21* | 0.05 |
| Diagnosis | η² = 0.305*** | η² = 0.040 | η² = 0.144*** |
| Progression to secondary fibrosis | 0.517*** | 0.0318 | 0.342** |
| Unprovoked thrombosis after diagnosis | 0.144 | -0.086 | 0.287** |
| Hemoglobin (g/dL) | -0.49*** | -0.26** | -0.29** |
| Hematocrit (%) | -0.50*** | -0.25*** | -0.30** |
| Total leukocyte count (×10⁹/L) | 0.27** | -0.14 | 0.36*** |
| Absolute neutrophil count (×10⁹/L) | 0.01 | -0.02 | 0.04 |
| Platelets (×10⁹/L) | -0.33*** | -0.10 | -0.16 |
| PB blasts (%) | 0.31*** | -0.08 | 0.33*** |
| BMA blasts (%) | 0.29** | -0.04 | 0.30*** |
| LDH (U/L) | 0.16 | -0.08 | 0.19* |
| HCV status | 0.11 | 0.20* | -0.07 |
| JAK2V617F allele burden (%) | -0.07 | 0.08 | -0.14 |
| Microvessel density | -0.05 | 0.03 | -0.06 |
| Reticulin grade | 0.35*** | 0.14 | 0.24** |
| Collagen grade | 0.31*** | 0.19* | 0.18* |

- **Legend and Notes:** Spearman’s/Pearson correlation analysis was used; only statistically significant correlations are marked with asterisks. p values: * p < 0.05, ** p < 0.01, *** p < 0.001. CD68⁺, CD163⁺: percentage of positive macrophages. Abbreviations: LDH: lactate dehydrogenase; HCV: hepatitis C virus; PB: peripheral blood; BMA: bone marrow aspirate.
